# Endoscopic placement of double-J ureteric stents in children as a treatment for primary obstructive megaureter

**DOI:** 10.4103/0974-7796.68860

**Published:** 2010

**Authors:** Daniel Carroll, Harish Chandran, Ashwini Joshi, Liam S. L. McCarthy, Karan Parashar

**Affiliations:** Department of Paediatric Urology, Birmingham Children’s Hospital NHS Trust, Steelhouse Lane, Birmingham, UK

**Keywords:** Double-J stent, megaureter, pediatric, ureteric stent

## Abstract

**Aim:**

To determine the efficacy and potential complications of double-J ureteric stents in the treatment of persistent or progressive primary obstructive megaureter in pediatric patients within our institution.

**Materials and Methods:**

A retrospective case-note review of all patients with double-J ureteric stents, between 1997 and 2004, was performed. In all, 38 stents were inserted in 31 patients aged between 2 months and 15 years of age. Complications and results of follow-up investigations and the need for follow-up investigations were recorded. Patients were followed up clinically and radiologically for a minimum of 2 years following stent insertion.

**Results:**

Endoscopic placement of double-J ureteric stents in childhood is straightforward and complications are uncommon (8/38 insertions). In non-resolving or progressive primary non-refluxing megaureter, double-J ureteric stenting alone is effective with resolution of primary non-refluxing megaureter in 66% of cases (25/38 insertions).

**Conclusions:**

Ureteric stenting provides an alternative to early surgery in patients with primary non-refluxing megaureter. The youngest patient in our series was 2 months old at the time of endoscopic ureteric double-J stent insertion. Endoscopic placement of ureteric double-J stents should be considered as a first-line treatment in the management of persistent or progressive non-refluxing megaureter leading to progressive hydronephrosis or pyonephrosis.

## INTRODUCTION

Ureteric tapering and reimplantation is the established treatment for primary obstructive megaureter that fails to resolve, or is associated with obstruction or pyonephrosis. For the past 10 years, we have used endoscopically placed double-J stents in children for the treatment of persistent or progressive non-refluxing megaureters.

The use of the endoscopically inserted indwelling double-J stent for achieving internal drainage of the ureters was first described over 20 years ago.[1] Since then, many other authors have reported their success, with the double-J stent in children as a safe and effective alternative to external drainage.[2–4] The double-J ureteric stent has been reported to allow for effective, reversible internal drainage of pediatric patients with primary non-refluxing megaureter (PNRM).[3,5] There are very few studies published on the use of double-J stents in the management of primary obstructive megaureters in children. The aim of our study is to report our experience over a 10-year period on the utility of ureteric stents in these patients.

## MATERIALS AND METHODS

A retrospective case-note review was performed of all patients who had double-J ureteric stent insertion for the treatment of primary obstructing megaureter at our institution. Between 1997 and 2004, 31 patients underwent endoscopic double-J stenting. Seven patients underwent re-stenting of the vesicoureteral junction (VUJ) as a secondary procedure.

All the patients underwent pre-operative assessment with renal tract ultrasound scanning and radionuclide renography. On ultrasound scan, the cross-sectional diameter of the retrovesical ureter and anteroposterior diameter of the renal pelvis in the transverse plane were determined.

The indications for endoscopic double-J stent insertion were either:

1. Progressive hydroureteronephrosis with a retrovesical ureter greater than 10 mm with an obstructive excretion pattern on dynamic radionuclide renography or
2. Evidence of impairment of function with differential renal function less than 40% with an obstructive excretion pattern on dynamic radionuclide renography or
3. The presence of pyonephrosis.

Patient demographics, complications and the results of follow-up investigations and outcomes for the patients including the subsequent need for ureteric reimplantation were recorded.

The ureteric stents used were Bard, American Scientific or Cook 3.0–4.7 Fr. multilength polyurethane double-J stents without valves. Such stents are provided with a guidewire and a 5-Fr. pusher and can be inserted transurethrally using a 9.5-Fr. operating cystoscope.

During stenting, the patients were maintained on antibiotic prophylaxis. Patients were reassessed 3 months following stent insertion on an outpatient basis, and all patients underwent ultrasongraphy (USS) of the renal tract. Patients were then electively readmitted for stent removal between 3 and 6 months following their outpatient review. Stents were removed endoscopically as a day-case procedure. A complete reassessment with routine renal tract USS was performed 3 months after stent removal. Evidence of increasing hydroureteronephrosis mandated dynamic radionuclide renography. Data were presented as descriptive statistics showing the median values and the range.

Patients underwent ureteral reimplantation if, at reassessment, the differential function was worsening. Seven patients underwent repeat double-J ureteric stenting for persistent hydroureteronephrosis following stent removal that did not fulfil the criteria for ureteric reimplantation.

## RESULTS

A total of 31 patients underwent double-J ureteric stenting for primary obstructive megaureter. Seven of these patients underwent repeat stenting. Cystoscopic insertion of the stent was possible in 36 out of 38 attempts, with an age range from 2 months to 15 years. In one patient in whom we were unable to insert a double-J stent at the first attempt (the patient was 5 years old), subsequent attempt at stenting a few weeks later was successful.

Our patients presented to us in one of three ways. The commonest presentation was in patients with antenatally diagnosed hydroureteronephrosis (20/31); the second largest group of patents presented with urinary tract infection and subsequent investigations for this (8/31); the remainder presented following an ultrasound scan for other reasons demonstrating an incidental finding (3/31).

Similarly, the indications for operative intervention were diverse. The commonest reason recorded in the notes was progressive hydroureteronephrosis with worsening hydronephrosis on serial ultrasound scans. In 20/31 patients, this was the reason recorded for ureteric stenting. A decrease in relative function to less than 40% on radionuclide scanning was the second most common indication for intervention (7/31 patients). Three patients underwent emergency stenting for pyonephrosis and one patient was stented for symptomatic VUJ obstruction.

Without clear indication for operative intervention, the patients were followed up with serial renal tract ultrasound scans. The median length of time that the patients were followed before an indication for intervention arose was between 6 and 12 months from the time of presentation, to a pediatric urologist (range 0–72 months). Eight out of 31 patients were followed up for less than 6 months; 14/31 patients were followed up between 6 and 12 months and 9/31 patients were followed for greater than 1 year before ureteric stenting was performed.

The median age at the time of stent insertion was 37 months with a range from 2 months to 15 years; the patient demographics are summarized in Table 1. Seven patients were stented on more than one occasion and one patient was stented with a solitary functioning kidney. Out of the 31 patients treated with double-J ureteric stenting, 11 required subsequent ureteric reimplantation, one because of intraoperative ureteric perforation by the stent.

**Table 1:** Summary of patient demographics of patients undergoing double-J ureteric stenting

Age (months)	Gender	Length of time stent *in situ* (months)
Median age (51)	8F:23M	Median (6)
Range (2–184)		0–6 months (n=20)
		6–12 months (n=16)
		13 months (n=2)

**Figure 1:**
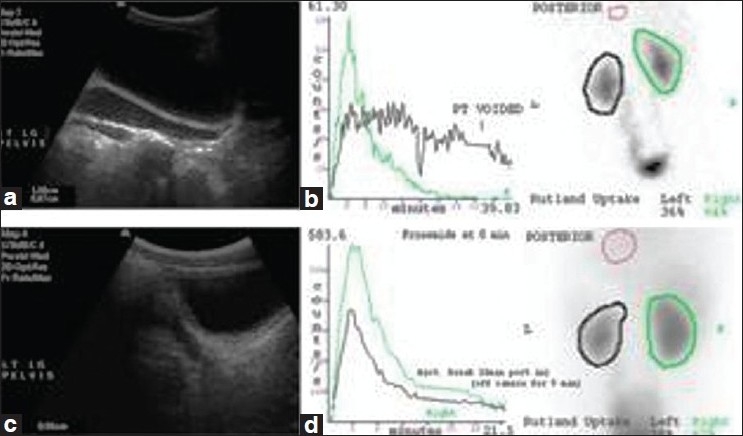
Pre- and post-op imaging of patient with PNRM - (a) USS prior to double-J stenting; (b) MAG-3 scan prior to double-J stenting; (c) USS following removal of double-J stent; (d) MAG-3 scan following removal of double-J stent

Follow-up was performed routinely at 3 months following ureteric stent insertion. All the patients were followed up for a minimum of 2 years following double-J stent removal or until 12 months following ureteric reimplantation. The median time of follow up was 39 months (range 24–110 months). The pre-operative and post-operative USS and MAG-3 scans in a typical patient treated successfully by double-J stenting are shown in Figure 1.

At follow-up, the patients were booked for elective stent removal which was planned for 6 months following insertion. Twelve patients had ureteric stents left for longer than 6 months with no adverse consequences. There were no episodes of symptomatic stone formation and no episodes of stent migration or difficulties in removing stents. Four patients had episodes of frank hematuria treated with tranexamic acid. Two patients had urinary tract infections and in one patient we were unable to insert a ureteric double-J stent for technical reasons. Failed stenting occurred on two occasions. In the first patient, a subsequent attempt to insert a DJ stent a few weeks later was successful. In one patient, an immediate ureteric reimplantation was performed because of ureteric perforation during stenting.

**Figure 2:**
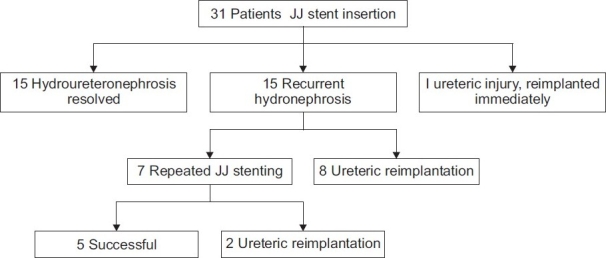
Results of JJ stenting

Of the patients who had persistent or progressive hydroureteronephrosis following stent removal, seven were successfully restented. Following a second period of stenting, five of these seven patients did not require ureteric reimplantation. A summary of our results is shown in Figure 2.

Of the patients who ultimately underwent ureteric reimplantation, five were males and six were females. The median age of patients requiring ureteric reimplantation was 51 months (range 2–110 months). The one patient with a solitary kidney required ureteric reimplantation as did the youngest patient in the series who was only 2 months at the time of ureteric stent insertion. Stenting, however, allowed reimplantation surgery to be deferred until the child was over 12 months of age.

## DISCUSSION

The pathophysiology of primary obstructive megaureter has not been adequately characterized. The obstruction at the vesico-ureteric junction is thought to be due to an adynamic segment of ureter. A number of different authors have attempted to characterize the histopathologic abnormalities seen in the adynamic segment. Dixon *et al*. described a thick sleeve of muscle around the distal ureter.[6] Lee *et al*. described abnormal collagen deposition[7] and Hertle and Nawrath described abnormal neuromodulation in obstructed megaureters.[8] Most recently, Kang *et al*. described abnormalities in the interstitial cells of Cajal in the obstructed megaureter.[9] These findings do not explain why in some patients the megaureter resolves spontaneously, but not in others.

The trend in the management of primary obstructive megaureter has changed significantly over the past 20 years. In 1989, Peters *et al*. reported that 89% of patients with obstructing megaureters underwent surgery by the age of 8 months.[10] This contrasts starkly with a publication from Keating *et al*. in the same year, which described conservative management in 87% of patients.[11] A natural history series from Great Ormond Street Hospital in 1994 demonstrated that the majority of megaureters can be managed conservatively.[12] In this series, Liu *et al*. reported that 34% of antenatally diagnosed megaureters had resolved spontaneously by the age of 3 years, 49% were persistently dilated and only 17% had been reimplanted.

The endoscopic placement of double-J ureteric stents in childhood is straightforward and in our series major complications were uncommon. To our knowledge, the first report in the literature to have described the use of double-J ureteric stents in infants with PNRM was over 10 years ago by Shenoy and Rance.[3] In their article, they described open rather than endoscopic insertion of these stents. Subsequently, Castagnetti reported only limited success with the use of double-J stents in 10 infants.[5] Most recently, Farrugia *et al*. have reported their experience in double-J stenting of 16 infants with primary obstructive megaureter.[13] They concluded that stenting was successful as the sole surgical treatment in 56% of cases but complications were commonplace, and that two-thirds of the infants required open stent insertion. Interestingly, we saw no episodes of stent migration in our series, which was reported as a common complication following open double-J stent insertion.

In our institution, all the stents were inserted endoscopically, even in infants, without resorting to open surgery. In this series, endoscopic stenting failed in only two patients, but was successfully placed at a subsequent attempt 3-4 weeks later in one of them. The other patient underwent immediate ureteric reimplantation following a ureteric perforation during attempted stenting.

It may be that vesico-ureteric junction dysfunction is similar to pelvi-ureteric junction dysfunction, and that only a proportion will require intervention.[11] Like hydronephrosis secondary to pelvi-ureteric junction dysfunction, hydroureteronephrosis secondary to vesico-ureteric junction dysfunction is managed conservatively. We have used failure of resolution of a megaureter greater than 1.0 cm and increasing hydronephrosis as an indication for ureteric stenting.

Primary non-refluxing megaureter resolves in almost half of all the patients after the first stent (15/31) and in 5/7 patients after a second attempt at stenting. Our practice has evolved over the last decade from ureteric reimplantation to give the patients with recurrent hydroureteronephrosis following stent removal, the opportunity for repeat stenting. These patients have been followed up for a minimum of 2 years following removal of the ureteric stent and have not required any further intervention. Failure of resolution or recurrent hydroureteronephrosis can be treated with further stenting. This was successful in 5/7 patients in our series. Furthermore, ureteric stenting is an alternative to early surgery and acts as a temporizing procedure in infants. Five of our patients were under 12 months of age at the time of stent insertion and only one of these patients has subsequently undergone ureteric reimplantation.

Endoscopic placement of double-J ureteric stents should be considered as a first line treatment in the management of persistent or progressive non-refluxing primary megaureter causing hydronephrosis or sepsis. This technique avoids unnecessary bladder surgery and the associated complications. Particularly in infants, in whom the risks associated with ureteric reimplantation are reported as being higher.[14,15]

A significant limitation of this study is that it is retrospective, and as such we were unable to control the indication for intervention. A natural history series needs to be done for patients with PNRM in a similar way to the data that have been collected for PUJ dysfunction. The authors would recommend a randomized controlled trial comparing conservative management of PNRM to stenting with rescue by randomization either to stenting or ureteric reimplantation.

## CONCLUSIONS

Our study suggests that ureteric stenting is a useful option in the management of primary obstructive megaureters requiring surgical intervention. The majority of patients who would have previously been subjected to ureteric reimplantation can be successfully managed by double-J stent insertion. In contrast to the experience of other authors,[3,13] we have been able to insert stents endoscopically in almost all children. This success is tempered by the knowledge that major complications can occur (ureteric perforation in one case) and that 11/31 patients in our series subsequently underwent ureteric reimplantation. In addition to this, patients treated with double-J stenting require longer and more intensive follow up than those treated with ureteric reimplantation. We believe that double-J stenting for primary obstructive megaureter is a useful tool in the armamentarium of the specialist pediatric urologist and, in particular, may be most useful as a temporizing measure in infants as an alternative to early surgery.
